# Interventions involving community health workers and their effect on cervical cancer screening uptake in sub-Saharan Africa: a systematic review

**DOI:** 10.1186/s12889-026-28109-2

**Published:** 2026-06-19

**Authors:** Sophie Hilda Lemoupa Makajio, Micol Murtas, Bruno Kenfack, Pierre Vassilakos, Nicole C. Schmidt, Patrick Petignat, Ania Wisniak

**Affiliations:** 1 Institute of Global Health, Faculty of Medicine, University of Geneva, Geneva, Switzerland; 2 Ministry of Public Health, Yaoundé, Cameroon; 3 Department of Gynecology, Obstetrics and Pediatrics, Geneva University Hospital, Geneva, Switzerland; 4 Department of Obstetrics, Gynecology and Maternal Health, Faculty of Medicine and Pharmaceutical Sciences, University of Dschang, Dschang, Cameroon; 5 Geneva Foundation for Medical Education and Research, Geneva, Switzerland; 6 Faculty of Social Sciences, Catholic University of Applied Sciences, Munich, Germany

**Keywords:** Cervical cancer screening, Community health workers, Sub-Saharan Africa

## Abstract

**Background:**

Sub-Saharan Africa (SSA) has the highest incidence and mortality rates of cervical cancer (CC), largely due to limited screening coverage. Community health workers (CHWs) are lay health providers who strengthen healthcare delivery; however, their influence on cervical cancer screening (CCS) uptake remains insufficiently explored. This systematic review evaluates the impact of CHW-involved interventions on CCS uptake in SSA.

**Methods:**

A systematic search of MEDLINE, EMBASE, and Cochrane databases was conducted in June 2023 and updated in May 2025. Studies were included if they: (1) were conducted in SSA among females eligible for CCS and published between January 2003 and May 2025; (2) compared CHW-based interventions with standard or non-CHW approaches; and (3) reported CCS uptake, linkage to triage and/or treatment after primary screening or attendance to follow-up visits. Only studies using a controlled design were included. A descriptive analysis was performed. PROSPERO registration: CRD42024495220.

**Results:**

Six studies met the inclusion criteria. Despite methodological limitations, all studies involved CHWs in health education and counseling. CHWs conducted home visits to recruit participants for CCS, rescheduled follow-up appointments, facilitated access to care, and provided childcare during screening attendance. Interventions involving CHWs significantly increased CCS uptake in three of the five studies assessing it as the primary outcome and improved follow-up attendance in the single study examining this indicator.

**Conclusion:**

The effectiveness of CHW-based interventions varies by type and context but shows potential to enhance CCS uptake in SSA.

## Introduction

Cervical cancer (CC) is the fourth most common cancer among women worldwide, with an estimated 660,000 new cases and 350,000 deaths reported in 2022 [1]. CC results from persistent infection with oncogenic human papilloma virus (HPV) [2], and sub-Saharan Africa (SSA) continues to experience the highest incidence and mortality rates [1]. To accelerate CC elimination, the World Health Organization (WHO) established 2030 targets: 90% of girls should be fully vaccinated against HPV before age 15 years, 70% of females screened with a high-performance test at ages 35 and 45 years, and 90% of those diagnosed with cervical disease receiving treatment [3].

Screening is fundamental to CC prevention because the disease progresses slowly. Early detection of precancerous lesions enables treatment before invasive cancer develops, reducing the need for resource-intensive care [4]. However, cervical cancer screening (CCS) uptake averages 19% in low-income countries compared with 63% in high-income countries [5]. A systematic review and meta-analysis of CCS coverage in SSA reported a pooled uptake of 12.9%, with considerable variation across countries [6]. Major barriers include limited access to screening services, low awareness and knowledge of CC, and socio-cultural constraints [7]. From the provider perspective, insufficient skills, inadequate equipment, and staff shortages impede effective CCS delivery [7]. The COVID-19 pandemic further reduced attendance at hospital-based screening units [8], highlighting the urgency of community-based strategies to improve coverage.

Community health workers (CHWs) are healthcare providers who reside within the communities they serve and generally possess less formal education and training than professional healthcare staff such as nurses and physicians [9]. In SSA, CHWs have been instrumental in strengthening the heath care workforce and improving access and outcomes across programs addressing immunization [10, 11], malaria [12] and HIV [13]. Nevertheless, evidence on the influence of CHWs on CCS uptake remains limited. This systematic review aimed to assess the effect of CHWs involvement on CCS uptake in SSA and related outcomes. Specifically, this review assessed whether CHW-led or CHW-supported interventions improve screening participation compared to standard care, identify which CHW activities are associated with increased uptake, and examine contextual factors influencing their effectiveness. By synthesizing available evidence, this study sought to clarify the potential contribution of CHWs to enhancing the reach, equity and effectiveness of cervical cancer screening programs in SSA. The review follows the Preferred Reporting Items for Systematic Reviews and Meta-Analysis (PRISMA) 2020 guidelines [14].

## Methods

### Search strategy

A systematic literature search was conducted in June 2023 and updated in May 2025 using MEDLINE, EMBASE, and the Cochrane Library (Cochrane Database of Systematic Reviews and Cochrane Central Register of Controlled Trials). Reference lists of eligible studies and relevant review articles were also screened for additional publications. Articles written in or translated into English or French and published between January 2003 and May 2025 were eligible. Unpublished studies were excluded.

The search strategy combined exploded MeSH and Emtree terms with free‑text synonyms for “cervical cancer” and “community health workers”; complete Boolean search strings for each database are provided in the Appendix 1. Eligible studies employed controlled designs evaluating CHW-based interventions, including randomized trials, cohort studies, before-after studies, and cross-sectional or case-control studies with a comparison group. Reviews, meta-analyses, commentaries, qualitative studies without quantitative components, study protocols, conference papers and abstracts were excluded.

### Eligibility criteria

Studies were eligible if participants were females living in SSA who met the criteria for CCS. Those involving females with a history of total hysterectomy or prior diagnosis were excluded. Interventions conducted at any level of the health care system – primary, secondary, tertiary- or within the community level, were eligible. Only studies conducted in SSA were included, based on the country information provided in the publication or its supplementary materials. Countries in SSA were defined according to the World Bank classification. The full list of countries is provided in Appendix 2.

CHWs were identified using the WHO definition. Equivalent designations, such as health auxiliaries, field workers, outreach workers, village health workers, lay health workers, community health aides or community promoters were accepted when descriptions aligned with the WHO definition.

Interventions involving CHWs were defined as activities led or supported by CHWs at any stage of CCS procedures, including population sensitization, recruitment of eligible females, specimen collection, screening test administration, and follow-up. Multifaceted strategies were eligible if CHWs participated in at least one component.

Comparators were CCS strategies that did not involve CHWs, including standard facility-based approaches or study interventions delivered without CHW participation.

The following outcomes were extracted for each study: the primary outcome was CCS uptake, defined as the proportion of eligible females who underwent screening during the study-defined period. Secondary outcomes included linkage to triage or treatment after initial screening, attendance at follow-up visits, and self-reported intention to undergo CCS.

### Study selection

Two reviewers (SLM and AW) independently screened article titles and abstracts using Covidence software (www.covidence.org) to identify studies meeting the eligibility criteria. Discrepancies in judgement were resolved through discussion with a third reviewer (MM).

### Risk of bias assessment

Risk of bias was assessed using the Cochrane Risk of Bias 2 (Rob2) tool for randomized controlled trials and the Risk of Bias in Non-randomized Studies of Interventions (Robins-I) tool for non-randomized studies. The Rob2 tool evaluates five domains: (1) bias arising from the randomization process; (2) bias due to deviations from the intended interventions; (3) bias due to missing outcome data; (4) bias in outcome measurement; and (5) bias in selection of the reported result. Each domain was rated as “low risk,” “some concerns,” or “high risk.” The (ROBINS-I) tool assesses seven domains: (1) bias due to confounding; (2) bias in participant selection; (3) bias in classification of interventions; (4) bias due to deviations from intended interventions; (5) bias due to missing data; (6) bias in outcome measurement; and (7) bias in selection of the reported result. Each domain was judged as “low risk,” “moderate risk,” “serious risk,” “critical risk,” or “no information.” Two reviewers (SLM and MM) independently evaluated each study, with a third reviewer (AW) resolving any disagreement.

### Data extraction

The following data were extracted from eligible studies: title, first author, publication year, study design, study period, country, setting (urban vs. rural setting; community- or facility-based, study population (age group), number of participants in intervention and control groups, type of intervention, comparator, number of females screened (primary outcome), and number followed up (secondary outcome).

Data extraction was undertaken independently by two reviewers. Discrepancies were resolved through discussion with a third reviewer. The final dataset was exported from Covidence in Excel format. Missing data were recorded and reported as such.

### Strategy for data synthesis

A narrative synthesis was conducted to summarize the findings of included studies. The synthesis was organized according to study characteristics: (1) type of intervention (e.g., door-to-door recruitment, community events), and (2) study outcomes (e.g., screening uptake, linkage to triage and/or treatment among screen-positive females).

### Review registration

The review protocol was retrospectively registered with the International Prospective Register of Systematic Reviews (PROSPERO) on 26 January 2024, CRD42024495220. At the time of submission, formal literature searches had begun; however, screening of search results against the inclusion criteria had not yet been performed.

## Results

**Fig. 1:**
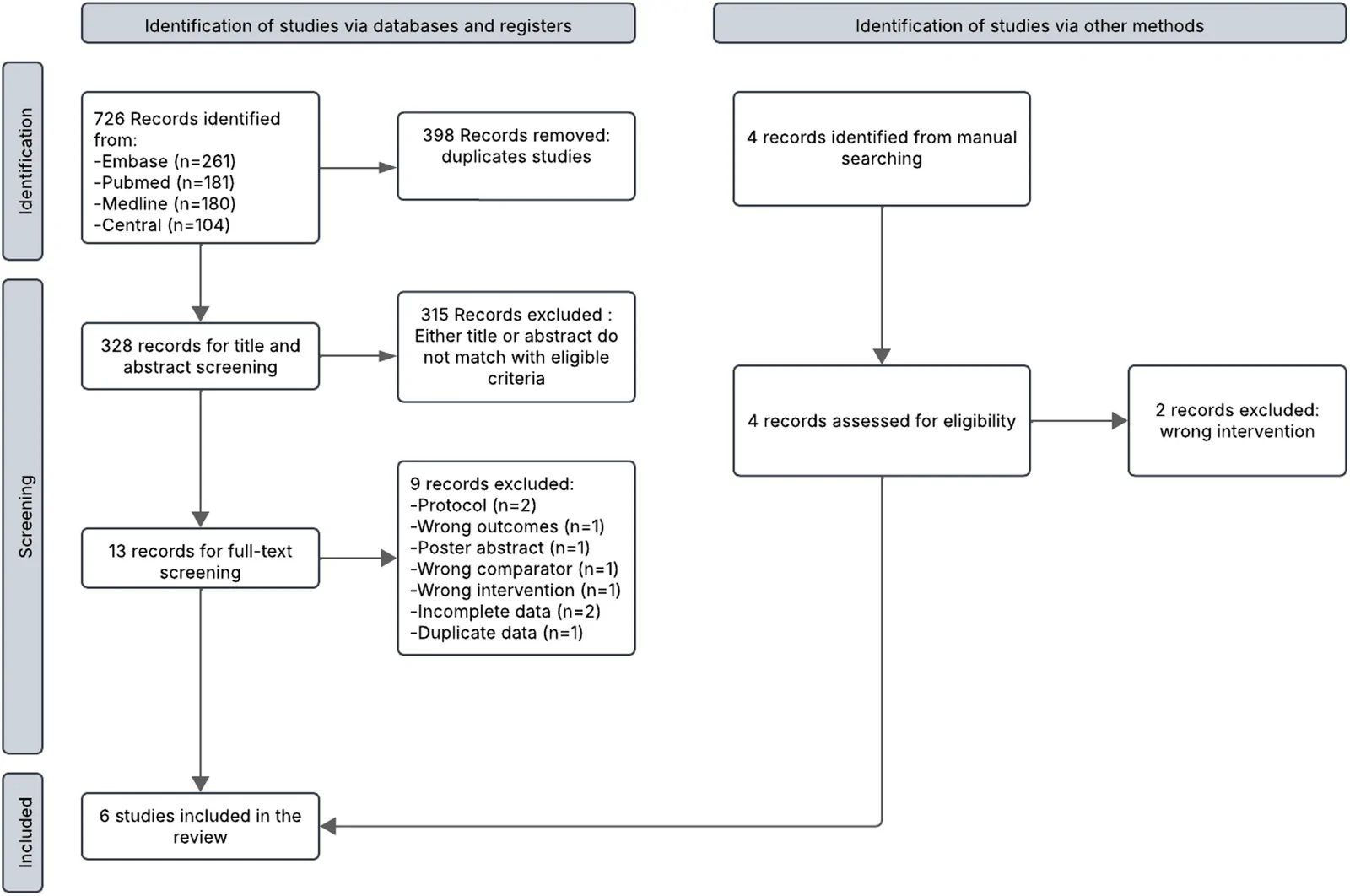
Study flow chart. The article selection process is illustrated according to the PRISMA 2020 flow. The literature search identified 726 records by electronic databases, and 4 records were identified by manual reference screening, yielding a total of 730 records. Six studies met the inclusion criteria and were retained for analysis

No additional studies meeting the inclusion criteria were published between June 2023 and May 2025. The initial search through electronic databases identified 726 records. After Covidence automatically removed 398 duplicates, 315 records were excluded during title and abstract screening because they did not meet the eligibility criteria. The remaining 13 records underwent full-text review, of which nine were excluded for the following reasons: study protocol (*n* = 2), poster abstract (*n* = 1), inappropriate outcomes (*n* = 1), unsuitable comparators (*n* = 1), irrelevant intervention (*n* = 1), incomplete data (*n* = 2), and duplicate data (*n* = 1). Manual reference searching identified four additional records, two of which were excluded after screening. In total, six studies met all inclusion criteria and were included in this review. The study selection process is illustrated in the PRISMA flow diagram (Fig. 1).

### Study characteristics

The six included studies involved 9,192 participants, with individual sample sizes ranging from 88 to 5,772 participants. The studies were published between 2003 and 2023. Two were conducted in South- Africa (33.3%), one in Central Africa: (Cameroon, 16.7%), one in West Africa (Nigeria, 16.7%), and two in East Africa (Tanzania and Kenya, 16.7% each). Five studies were community based, and one was facility based. Settings included urban (*n* = 1, 16.7%), rural settings (*n* = 2, 33.3%), peri-urban settings (*n* = 2, 33.3%) and mixed urban-rural settings (*n* = 1, 16.7%). Two studies (33.3%) used before-after designs, two were randomized controlled trials (33.3%) and two were prospective cohort studies (33.3.%). Participants were females aged 20–65 years (Table  1).

**Table 1:** Profile of included studies and key findings

Author, Country, Year,	Study design, setting, study duration, sample size	Population	Intervention (I) /Comparator (C)	Outcome/Results
Goldhaber-Fiebert et al., South-Africa (2009) [15]	Prospective cohort study Community-based Peri-urban Duration: 2 years Sample size: 6 months: 1,003 12 months: 1,992 24 months: 1,969 36 months: 808	Females aged 35–65 years	I: Home visits to reschedule appointments and encourage attendance with CHW visits repeated until return is achieved, or further participation is declined. C: Follow-up without CHWs’ visit	*Outcome*: Attendance of scheduled follow-up visits at 6, 12, 24 and 36 months. Outcome ascertainment: through study appointments database and CHWs records *Results*: 6 months: I:907/1,003 (90%) C:740/1,003 (74%) 12 months: I: 1,739/1,992 (87%) C: 1,096/1,992 (55%) 24 months: I: 1,518/1,969 (77%) C: 944/1,969 (48%) 36 months: I:647/808 (80%) C: 453/808 (56%)
Mbach u et al., Nigeria (2017) [16]	Before and after design Community-based in Anglican churches Urban Duration: unknown Sample size: (*n* = 300)	Females aged 21 years	I: Peer health education on CC attended during 3 to 6 sessions.	*Outcome*: CCS uptake. Outcome ascertainment: through interview *Results*: Before: 30/285 (10.5%) After: 49/283 (17.3%) Significant increase in CC screening of + 6.8% (*p* = 0.02).
Mboineki et al., Tanzania (2022) [17]	Randomized controlled trial Community-based Urban Duration: 6 months Sample size: I: 44 C: 44	Females aged 21–50	I: Health education by CHWs about CC and CCS; counselling for females who did not undergo screening, navigation support and childcare assistance for mothers attending screening. C: Health education on CC and its prevention through the ongoing national CCS awareness campaign and other channels	*Outcomes*: -CCS uptake at 6 months follow-up. -CCS intention at 3- and 6-months follow-up Outcome ascertainment: through interview *Results*: CCS uptake: 3 months follow-up: I: 29/44 (65.9%) C: 1/44 (2.3%) 6 months follow-up: I: 32/44 (72.7%) C: 1/44 (2.3%) CCS intention score: Baseline (T0): No significant difference in CCS intention between I (86.1 ± 17.4) and C (86.6 ± 0.23.5) 3 months follow-up (T1): I: 141.7 ± 7.5 C: 86.6 ± 23.5 6 months follow-up (T2): I:145.2 ± 5.3 C:86.6 ± 23.5 (*p* < 0.001)
Pham et al., Cameroon (2022) [18]	Before and after design Community-based Rural Duration:18 months Sample size: (*n* = 1940)	Females aged 30–49 years	I: CHW-led: door-to-door sensitization, invitation vouchers for CCS given to eligible females C: CIC led: radio announcement, street banners, flyers, posters in public places	*Outcome*: CCS uptake Outcome ascertainment: screening centre records *Results*: I: 584/1,940 (30.1%) C: 1356/1,940 (69.9%) Proportion of screened females living in rural areas increased from 12.1% to 61.4% among all females screened in CIC-led and CHW-led respectively.
Rosser et al., Kenya (2015) [19]	Randomized controlled trial Facility-based Rural Duration: 5 months Sample size: I: 207 C: 212	Females aged ≥ 23 years	I: 30-minute interactive talk about CC by CHWs C: No health talk	*Outcome*: CCS uptake Outcome ascertainment: through interview and health facilities records *Results*: I: 122/207 (58.9%) C: 129/212 (60.9%) No statistically significant difference between the two groups
Tum et al., South-Africa (2013) [20]	Before and after design, Community-based Urban Duration: 8 months Sample size: 673	Females aged ≥ 20 years	I: Community health education on CC and breast cancer, home visits and encouraging uptake of CC screening	*Outcome*: CCS uptake Outcome ascertainment: records of the Cancer Nursing Community of Practice and available statistics *Result*: Before: 1.3% After: 21/673 (3.1%)

*CC* Cervical Cancer, *CCS* Cervical Cancer Screening, *CHWs* Community Health Workers, *CIC* Communication Information Channels, *I* Intervention, *C* Control, *PNLav* Peer-Led Navigation

### Type of interventions carried out by CHWs

Across all included studies, CHWs participated in health education and counselling [15–20]. In four studies, CHWs performed visits aiming to recruit participants [18, 20], to reschedule follow-up [15], assist with navigation to healthcare facilities [17] or provide childcare while mothers attended screening sessions [17].

### Study outcomes

Five studies [16–20] assessed the effect of CHW interventions on CCS uptake as the primary outcome, and one [15] evaluated attendance at scheduled follow-ups. CHW involvement improved CCS uptake from 10.5% to 17.3% in Nigeria [16], and from 12.1% to 61.4% of females in rural Cameroon [18]. In Tanzania, 73% of females who received a peer-led navigation (PLNav) intervention underwent CCS compared with 2.3% in the control group [17]. In contrast, CHWs interventions in Kenya [19] and South Africa [20] showed no significant difference in CCS uptake. In another study in South Africa [15], 74%, 55%, 48%, and 56% of females attended their scheduled 6-, 12-, 24-, and 36-months appointments without CHWs’ contact, versus 90%, 83%, 74%, and 77% respectively after two CHW’s visits.

The Tanzanian study also reported a PLNav intervention’s effect on CCS intention as a secondary outcome. Screening intention was determined by composite scores of CCS attitude, subjective norms, and perceived behavioral control. At baseline, intention did not differ between groups; however at 3 and 6 months after the PLNav, the intervention group showed significantly higher intention scores (T0 = 86.1 ± 17.4, T1 = 141±7.5, and T2 = 145.2 ± 5.3) compared with the control group intention score (T0 = 85.4 ± 23.9, T1 = 86.8 ± 23.5, and T2 = 86.6 ± 23.5) [F(1,43) = 106.6, *p* < 0.01] [17].

### Quality assessment of the studies

**Fig. 2:**
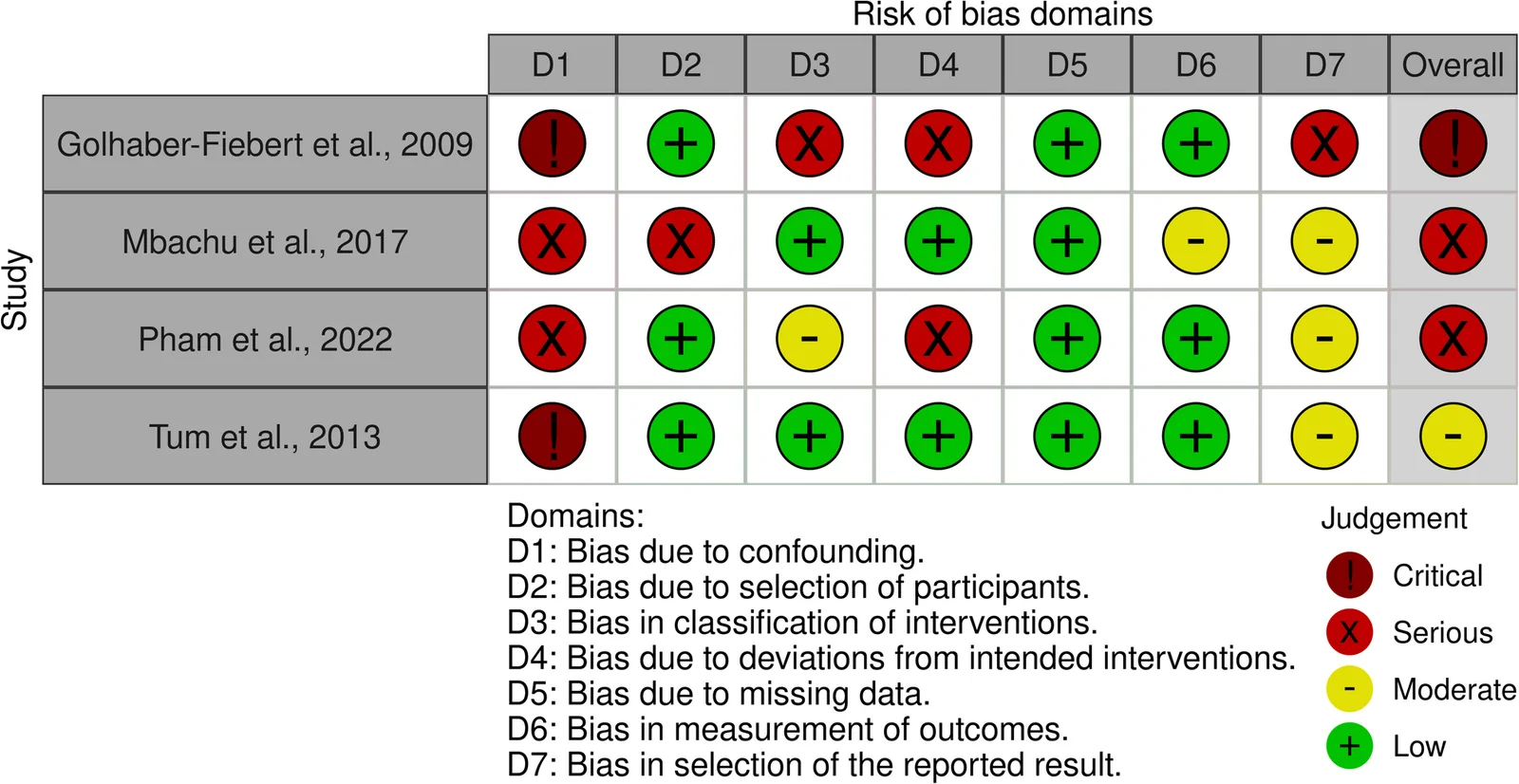
Analysis of risk of bias among the included studies using the ROBINS-I tool

Among the non-randomized studies, one demonstrated an overall critical risk of bias, two showed an overall serious risk, and one presented a moderate risk of bias ( Fig. 2).

The randomized controlled trial conducted in Tanzania exhibited an overall moderate risk of bias, whereas the trial performed in Kenya demonstrated a low risk of bias (Table 2).

**Table 2:** Quality assessment of included studies using the RoB 2 tool

	Cochrane Risk of Bias 2					
	Randomization process	Deviations from the intended interventions	Missing out come data	Outcome measurement	Selection of the reported result	Overall risk of bias assessment
Mboineki et al., 2022	Low	Low	Low	High	Some concerns	Moderate
Rosser et al., 2015	Low	Low	Low	Low	Some concerns	Low

## Discussion

This systematic review examined the effects of CHW interventions on CCS uptake in SSA. Six studies met the inclusion criteria. All studies involved CHWs in health education and counseling [15–20]. Other activities included: home visits [18, 20], navigation to care [17], invitation for screening [18] and follow-up [15]. Interventions involving CHWs significantly increased CCS uptake in three of the five studies assessing it as the primary outcome [16–18] and improved follow-up attendance in the single study examining this indicator [15].

The included studies were implemented either as standalone or multi-component strategies. In all studies, CHWs delivered health education on CC and screening across diverse settings, including health facilities [19], churches [16] and within homes or communities [15, 17, 18, 20]. The outcomes of these interventions varied by context. Health education, alone or combined with other strategies, effectively increased CCS uptake in urban Nigeria [16], Tanzania [17], and rural Cameroon [18]. However, a 30-minute interactive education session in rural Kenya did not yield a statistically significant improvement [19]. In Iran, CHW-led education interventions based on protection motivation theory enhanced Pap test use [21], and in the United States, a CHW-led intervention among Hispanic females increased Pap screening rates, knowledge, and self-efficacy [22]. These findings indicate that CHW-led education effectiveness may depend on geographic, infrastructural, individual, or cultural factors.

The association between knowledge and screening uptake is complex. High awareness does not always translate to participation. Among female health workers in Nigeria, 98% reported awareness, yet 19.8% had been screened, citing barriers such as fear of complications and high costs [23]. Similarly, among mothers attending antenatal and postnatal clinics, awareness was moderate (59.8%) but screening remained low (36.1%) [24]. Barriers to CC screening in SSA are well documented and include logistical, cultural, and systemic factors [7, 25]. To address these challenges, CHWs must possess not only biomedical knowledge but also strong communication and motivational skills. For instance, motivational interviewing- delivered face-to-face or by telephone- has improved screening uptake for breast and cervical cancers screening [26].

Although initial training is essential, continuous assessment and supervision are equally critical for maintaining CHW performance [27]. Studies in Nigeria [16] and Tanzania [17] did not specify whether CHW competencies were assessed before and after training, or describe supervision during implementation. Financial motivation also influences CHW performance [27, 28 ]. In Cameroon, CHWs were paid per recruited participant [18], whereas in Nigeria [16] and South-Africa [20], they received only transport reimbursement during training. Despite differing incentive structures, all three interventions improved screening uptake. This suggests that factors beyond financial incentives—such as training quality, community trust, supervision, and intrinsic motivation—may be crucial in driving CHW effectiveness [29]. The influence of incentives may also depend on the broader CHW financing and employment systems.

Home visits and community sensitization are increasingly used for CCS in SSA. CHWs often conduct door-to-door enumeration and education before screening campaigns [30–32], fostering trust and open communication. Home-visits allowed to effectively rescheduled appointments and encourage attendance among participants lost to follow-up [15], while PLNav improved CCS uptake [17]. In Cameroon, invitation vouchers distributed after door-to-door sensitization increased screening uptake among rural females [18]. In the EMA study in Argentina, home-based self-collection for HPV testing provided by CHWs during home visits produced a four-fold increase in CCS compared with invitation vouchers issued after health education sessions [33]. A multicomponent intervention comprising health education, monthly telephone follow-ups, and navigation assistance also improved CCS uptake among South-Asian women in Hong-Kong [34].

None of the included studies formally assessed CHW workload in CC prevention or reported whether the CC-related activities were integrated with other health programs. However, a time-and-motion study in Malawi demonstrated that integrating CC prevention activities with other health program responsibilities is feasible, when CHWs receive close supervision and effective time management support [35].

This review has several limitations. Despite identifying many articles, few met the inclusion criteria, limiting analysis to a descriptive synthesis. The risk-of-bias assessment revealed multiple methodological concerns. All the non-randomized studies included were judged to have serious to critical bias due to confounding which may have led to overestimation or underestimation of the true effect of the intervention [15, 16, 18, 20]. This is particularly important given the observational nature of these studies and the likelihood that key confounders were not fully accounted for. The two included randomized controlled trials were judged to have ‘some concerns’ regarding selection of the reported results, reflecting limited information on pre-specified outcomes and analyses [17, 19]. This raises the possibility of selective reporting, which may have influenced the results.

Nonetheless, a strength of this review is the inclusion of studies comparing CHW-led interventions to standard procedures or interventions without CHWs, allowing evaluation of their specific contributions. The findings underscore the need for robust comparative studies to clarify CHWs’ impact on CCS uptake in SSA.

While CHW-based interventions show potential to improve CCS [15–18], their effectiveness appears to depend on contextual factors including training, supervision, intrinsic motivation and health system [27, 29]. Task shifting to CHWs can therefore be a viable strategy for addressing health personnel shortage, but only when implemented with adequate resources and oversight. These results highlight the importance of developing well-structured community health workforce policies that ensure proper training, supervision, and integration of CHWs into formal health systems in SSA.

## Conclusions

In SSA, CHWs primarily support CC screening through health education, home visits, invitation for screening, navigation to care, and follow-up. The effectiveness of these interventions varies by type and setting. While evidence suggests that CHWs can enhance screening uptake, further rigorous research is needed to confirm these effects and elucidate underlying mechanisms.

## Data Availability

Data is provided within the manuscript or supplementary information files.

## Appendix

### Appendix 1

#### Search strategy

The following searches were conducted for the key terms “cervical cancer” and “community health workers” including their variations and equivalent MeSH terms.

MEDLINE:

- ("uterine cervical neoplasms"[MeSH Terms] OR ("cervi*"[All Fields] AND "cancer"[All Fields]) OR ("cervi*"[All Fields] AND "neoplas*"[All Fields]) OR ("cervi*"[All Fields] AND "dysplas*"[All Fields]))
- AND ("community health workers"[MeSH Terms] OR "community health worker*"[All Fields] OR "outreach worker*"[All Fields] OR "field worker*"[All Fields] OR “health auxiliar*”[All Fields])

EMBASE:

- ('cervical cancer'/exp OR ('cervi*' AND 'cancer') OR ('cervi*' AND 'neoplas*') OR ('cervi*' AND 'dysplas*'))
- AND ('community health worker*' OR 'outreach worker*' OR 'field worker*' OR 'health auxiliar*')

Cochrane Library:

-  #1 MeSH descriptor: [Uterine Cervical Neoplasms] explode all trees
- #2 MeSH descriptor: [Uterine Cervical Dysplasia] explode all trees
- #3 (cervi* NEAR (cancer or neoplas* or dysplas*)) (Word variations have been searched)
- #4 #1 OR #2 OR #3
- #5 MeSH descriptor: [Community Health Workers] explode all trees
- #6 (community NEXT health NEXT worker*) OR (community NEXT worker*) OR (outreach NEXT worker*) OR (health NEXT auxiliar*) OR (field NEXT worker*) (Word variations have been searched)
- #7 #5 OR #6
- #8 #4 AND #7 with Cochrane Library publication date Between Jan 2003 and May 2023

### Appendix 2

#### Participants/population

Eligibility for this review was based on the World Bank list of countries classified as part of Sub-Saharan Africa. These include Angola, Benin, Botswana, Burkina Faso, Burundi, Cabo Verde, Cameroon, Central African Republic, Chad, Comoros, Congo, Democratic Republic of, Congo, Republic of Cote d'Ivoire, Equatorial Guinea, Eritrea, Eswatini (Formerly Known as Swaziland), Ethiopia, Gabon, Gambia, Ghana, Guinea, Guinea-Bissau, Kenya, Lesotho, Liberia, Madagascar, Malawi, Mali, Mauritania, Mauritius, Mozambique, Namibia, Niger, Nigeria, Rwanda, Sao Tome and Principe, Senegal, Seychelles, Sierra Leone, Somalia, South Africa, South Sudan, Sudan, Tanzania, Togo, Uganda, Zambia, Zimbabwe.

## Abbreviations

CC: Cervical Cancer
CCS: Cervical Cancer Screening
CHW: Community Health Worker
PLNav: Peer Led Navigation
RoB: Risk of Bias
SSA: Sub-Saharan Africa
WHO: World Health Organization
PRISMA: Preferred Reporting Items for Systematic Reviews and Meta-Analysis
